# Audiovisual integration in reading among school-aged children: Evidence from combined fMRI and EEG

**DOI:** 10.1016/j.dcn.2026.101729

**Published:** 2026-04-28

**Authors:** Jinglei Ren, Haiwa Wang, Nicole Landi, Marc F. Joanisse, Vincent Gracco, Dan Kleinman, Kelly Mahaffy, Aahana Bajracharya, Roeland Hancock, Kenneth Pugh, Fumiko Hoeft

**Affiliations:** a School of Medicine, Yale University, United States; b Department of Psychology, University of Connecticut, United States; c Department of Developmental and Behavioral Pediatrics, Shanghai Children's Medical Center, China; d Department of Psychology, The University of Western Ontario, Canada; e BrainWorks, Wu Tsai Institute, United States; f Haskins Laboratories, United States

**Keywords:** Audiovisual integration, Reading development, Multimodal, FMRI, EEG

## Abstract

Reading fluency depends on rapid integration of print and speech, yet the spatiotemporal mechanisms supporting this integration in school aged children remain poorly understood. This study combined fMRI and EEG to examine AV integration (i.e., additive enhancement and congruency effects) during single- and multi-letter detection tasks in 7–10-year-old children (*N* = 56). Multiple neural indices were examined to capture complementary spatial and temporal aspects of AV integration. fMRI results revealed additive enhancement in bilateral STG and Heschl’s gyrus, congruency effects in the left IFG and left STG and increased functional connectivity between left IFG and STG during incongruent trials. EEG showed convergent temporal dynamics, with AV-related enhancement in P200 amplitude (190–230 ms) and theta-band (4–7 Hz) power over left frontotemporal electrodes. Individual differences analyses showed that additive enhancement in left STG was associated with higher spelling (*β* = 0.28, *p* = 0.034). Congruency effects in left STG (*β* = 0.31, *p* = 0.018) and left IFG (*β* = 0.38, *p* = 0.004), as well as IFG–STG connectivity (*β* = 0.34, *p* = 0.011), were linked to higher reading efficiency. Larger P200 amplitude (*β* = 0.32, *p* = 0.015) and greater theta power (*β* = 0.31, *p* = 0.021; *β* = 0.28, *p* = 0.034) were associated with higher spelling and fluency, respectively. Findings delineate complementary sensory-convergence and conflict-monitoring pathways for print–speech integration and show that integrating spatial and temporal neural indices improves explanation of reading skills in school-aged children.

## Highlights

• Combined fMRI + EEG reveal spatial-temporal markers of print–speech integration.
• Left STG and IFG jointly support additive enhancement and congruency effects.
• Theta power and P200 amplitude index rapid audiovisual binding in reading.
• Different neural indices uniquely predict diverse reading skills.
• Multimodal models explain more variance in reading skills than unimodal ones.

## Graphical Abstract

**Figure:**
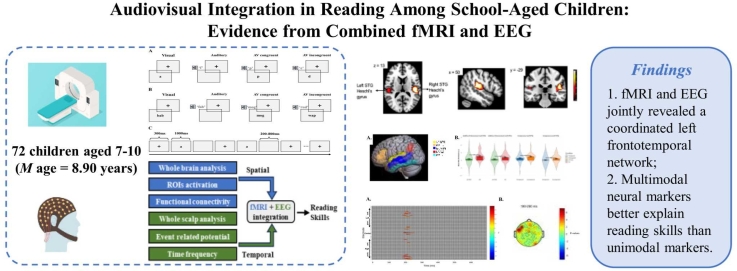

Learning to read is a complex process that requires the coordination of multiple cognitive and linguistic systems (e.g., Perfetti, 1986; Tunmer and Hoover, 2017). Unlike spoken language, which is typically acquired through exposure, reading is a culturally invented skill that depends on formal instruction and practice. In alphabetic languages, learning to read requires children to map visual letters onto their corresponding speech sounds, linking orthographic and phonological codes. This process relies on the brain’s ability to bind visual and auditory information, which through reading experience becomes specialized for print–speech mapping (Ehri, 1997, Ehri, 2005, Coltheart, 2005, Reitsma, 1983, Gonzalez-Frey and Ehri, 2021). Through repeated decoding practice, children gradually establish a mental lexicon of familiar word forms that can be accessed directly by sight. Even skilled readers rely on grapheme–phoneme mappings particularly when decoding novel or low-frequency forms (Coltheart, 2005, Ehri, 2005; Siegelman et al., 2020). Efficient, automatic coordination between print and speech is a hallmark of skilled reading (Blomert and Froyen, 2010, Richlan, 2019). When this print–speech integration is disrupted, the formation of stable associations breaks down, which is a core deficit observed in developmental reading disability (Blau et al., 2009, Froyen et al., 2011). To understand how skilled readers achieve this coordination and why some learners struggle with it, it is crucial to investigate the neural mechanisms underlying print–speech integration.

Previous research has examined how the brain integrates information during reading and related language tasks (e.g., Karipidis et al., 2017, 2021; Raij et al., 2000; van Atteveldt et al., 2004; Beauchamp et al., 2004). A primary approach has been used to compare neural and behavioral responses to audiovisual (AV) stimuli with responses to unimodal auditory-only or visual-only stimuli (Raij et al., 2000, van Atteveldt et al., 2004). This design assesses whether simultaneous presentation of auditory and visual inputs yields ***additive enhancement***, i.e., increased neural activation or improved behavioral performance relative to the combined (sum or mean) unimodal responses. Additive enhancement is thought to index the efficiency and strength of cross-modal integration, providing a measure of how effectively the brain combines print and speech information. A complementary line of research has focused on ***congruency effects***, comparing neural responses to congruent versus incongruent AV pairings (e.g., matching vs. mismatching letter–speech sound combinations) to probe the evaluation of cross-modal consistency and reliability (van Atteveldt et al., 2007). Congruent AV input typically facilitates performance, producing faster response times and higher accuracy than incongruent input in letters, words, nonwords, Chinese characters, pictures, animal vocalizations, and newly learned symbol–sound associations (Holcomb et al., 2005, Quémart et al., 2018, Xie et al., 2017, Blackford et al., 2012, Chen and Spence, 2018, Collignon et al., 2008, Hocking and Price, 2009, Jost et al., 2014). While congruency paradigms illuminate how the brain detects and resolves conflicting sensory information, additive enhancement paradigms more directly capture the integrative gain that arises when auditory and visual inputs are successfully combined. Together, these findings highlight additive enhancement and congruency effects as key neural markers of successful audiovisual (AV) integration (i.e., the neural gain arising from binding auditory and visual inputs into a unified representation) during reading in general.

Neuroimaging studies have identified a distributed network of regions supporting AV integration in reading. Functional Magnetic Resonance Imaging (fMRI) studies consistently implicates the bilateral posterior superior and middle temporal gyri/sulci (STG, MTG/STS), left fusiform gyrus (FG), left inferior frontal gyrus (IFG), Heschl’s gyrus (HG), and planum temporale (PT) as core regions mapping letters to their associated speech sounds, although precise activation patterns vary across tasks and developmental levels (Blomert, 2011, Brem et al., 2010, Hashimoto and Sakai, 2004, Herdman et al., 2006, Karipidis et al., 2021, Raij et al., 2000, van Atteveldt et al., 2004; di Pietro et al., 2023; Beauchamp et al., 2004; Calvert, 2001; Taylor et al., 2006; Kronschnabel et al., 2014). In addition, the supramarginal gyrus (SMG) and the angular gyrus (AG) in the parietal lobe have been shown to be involved in accessing phonological representations for written words and letter strings in both typical (Church et al., 2011) and struggling readers, though activation in these regions is often reduced or atypical in individuals with reading difficulties (Vandermosten et al., 2016, Xu et al., 2018, Pugh et al., 2000).

More recent work has shifted from identifying isolated loci toward examining how large-scale circuits support AV integration during reading (Rüsseler et al., 2018, Ye et al., 2017, Wang et al., 2020, Yang et al., 2020). Converging developmental and adult findings suggest that efficient reading depends on dynamic coordination among sensory and higher-order association cortices. Functional connectivity among the occipitotemporal cortex, STG, IFG and default mode networks appears to facilitate the integration of visual–orthographic and auditory–phonological information. Disruptions in these connections, consistently observed in poor readers across languages, indicate atypical communication between neural systems involved in print and speech processing (Ye et al., 2017, Rüsseler et al., 2018, Wang et al., 2020, Yang et al., 2020). Together, these findings highlight that AV integration depends not only on the activation of discrete cortical regions, but also on the integrity and efficiency of the networks connecting them. These networks are essential for establishing and consolidating print–speech associations throughout reading development.

Electroencephalography (EEG) complements the spatial precision of fMRI by revealing the temporal dynamics of AV integration with millisecond resolution. Recent studies have primarily focused on additive enhancement effect as a key neural signature of successful print–speech integration. Additive enhancement typically emerges within the first 200 ms after stimulus onset and is reflected in early ERP components such as the N1 (around 150–250 ms), which indexes orthographic and speech-sound processing and becomes increasingly specialized with reading development (Maurer et al., 2006, Brem et al., 2010, Zhao et al., 2014, Kaganovich et al., 2014). AV stimuli often elicit a larger N1 amplitude than unimodal inputs, indicating enhanced neural binding of visual and auditory information (Brandwein et al., 2011). At slightly later processing stages, additive enhancement is observed in the P2 component (around 200–300 ms), which reflects higher-order perceptual and attentional processes involved in integrating visual and auditory inputs (Duncan et al., 2009, Holcomb et al., 2005). AV stimuli typically elicit a reduced P2 amplitude compared with the summed A + V response, indicating predictive facilitation, whereby visual input primes and accelerates the processing of its corresponding auditory counterpart (Kaganovich et al., 2014, Treille et al., 2017). The congruency effect is most evident in the N170 (around 150–200 ms) and later ERP components, revealing the brain’s sensitivity to mismatches between print and speech inputs (Brefczynski-Lewis et al., 2009, van Atteveldt et al., 2007). Behaviorally, congruent AV stimuli elicit faster and more accurate performance than incongruent or unimodal stimuli, suggesting that consistent print–speech mapping facilitates more efficient phonological and lexical processing (Holcomb et al., 2005, Hocking and Price, 2009, Quémart et al., 2018, Xie et al., 2017, Pilling, 2009).

Time–frequency analysis, in turn, decomposes the same EEG signal into oscillatory components across time and frequency, quantifying both phase-locked (evoked) and non–phase-locked (induced) neural dynamics that contribute to multisensory processing (Makeig et al., 2002, Tallon-Baudry and Bertrand, 1999). Neural oscillations in the theta (4–7 Hz), beta (13–30 Hz), and gamma (30–80 Hz) ranges, in both power and phase dynamics, have been consistently linked to multisensory integration and the development of reading skills. These rhythms typically show greater power in AV conditions than in unimodal stimulation, reflecting enhanced cross-modal coupling and neural synchronization during integration (Giraud and Poeppel, 2012, Cainelli et al., 2023). Frontal theta oscillations are thought to facilitate the temporal alignment of auditory and visual streams, thereby enhancing phonological sensitivity (Brookshire et al., 2021, Attaheri et al., 2022, Attaheri et al., 2024). Gamma activity reflects the rapid binding of orthographic and phonological representations across sensory cortices (Friese et al., 2016), whereas beta synchrony contributes to top-down predictive control during later stages of integration (Hipp et al., 2011, Senkowski et al., 2008). Recent reading research increasingly integrates ERP and time–frequency approaches, offering a comprehensive characterization of how transient and rhythmic neural activities jointly support the temporal coordination of cognitive process (e.g., Zhang et al., 2025).

Despite considerable progress, important questions remain about how AV integration unfolds across space and time during reading. Most prior studies have relied on a single modality, either fMRI or EEG, capturing only part of the spatiotemporal dynamics that support print–speech integration. A notable exception is Kronschnabel et al. (2014), who combined simultaneous EEG and fMRI to examine letter-speech sound integration in adolescents with and without dyslexia, demonstrating the value of multimodal neuroimaging for understanding print-speech processing. However, this approach has not been extended to younger school-aged children (7–10 years), a critical developmental window during which children transition from effortful decoding to fluent reading. Furthermore, even multimodal studies have typically evaluated only one neural index per modality (e.g., regional activation in fMRI or ERP amplitude in EEG), making it difficult to determine which neural processes are most relevant to reading ability, and have not tested whether combining modalities improves prediction of individual differences in reading skills. fMRI offers high spatial precision to identify where cortical regions are engaged in print–speech integration, whereas EEG provides millisecond-level temporal sensitivity to reveal when integration occurs. Consequently, our understanding of print–speech integration remains incomplete. This represents a critical mechanistic gap, as fluent reading depends on the rapid temporal coordination between visual and auditory systems, supported by dynamic interactions across distributed brain networks. Addressing this limitation requires integrating the complementary strengths of fMRI and EEG, incorporating multiple neural indices, and employing a multimodal framework to achieve a more complete understanding of the mechanisms underlying AV integration and their relationship to reading development.

## 1 The present study

The present study addressed key gaps in the literature on AV integration in reading and its relation to reading ability by combining fMRI and EEG within the same participants. fMRI analyses included whole-brain activation, anatomically defined region-of-interest (ROI), and functional connectivity analyses to identify cortical regions and network-level interactions supporting AV integration. EEG analyses were conducted in both the time domain (whole-scalp, ERPs) and frequency domain (time–frequency analysis) to capture rapid neural responses and sustained oscillatory activity associated with multisensory processing. Together, these approaches provided a comprehensive spatiotemporal characterization of how the brain binds visual and auditory information during reading, linking electrophysiological signatures to their cortical generators. A second goal was to focus on an understudied developmental window. Most prior research has examined beginning readers or adults, leaving a gap in understanding children in the intermediate years of schooling. The ages of 7–10 represent a critical transition period: children have mastered basic decoding but are still developing reading fluency and automaticity. Examining AV integration during this stage offers insight into how neural mechanisms of print–speech mapping support the shift from effortful decoding to efficient word recognition and contribute to individual differences in reading ability. Finally, while prior studies have focused primarily on single grapheme–phoneme correspondences, little is known about how children integrate multi-letter orthographic units with their corresponding sounds. Investigating responses to short, pronounceable nonwords allows examination of word-like orthographic–phonological integration while minimizing semantic influences, thereby isolating the core neural mechanisms that support skilled reading.

Overall, this study aimed to 1) characterize the neural mechanisms supporting AV integration during this critical developmental period by combining fMRI and EEG to capture both the spatial organization and temporal dynamics of integration; 2) examine how neural markers, including fMRI activation and connectivity, ERP responses, and oscillatory power, relate to individual differences in reading efficiency, fluency, and spelling. These behavioral measures represent complementary dimensions of skilled reading: efficiency and fluency reflect the speed and automaticity of print–speech integration, whereas spelling reflects the quality of underlying orthographic representations; and 3) test whether integrating fMRI and EEG measures within a multimodal framework improves the explanation of individual differences in reading skills compared with unimodal models. Together, these aims provide a comprehensive account of how coordinated brain processes jointly support the development of fluent and efficient reading.

## 2 Methods

### 2.1 Participants

Children aged 7–10 were eligible to participate in the study if they were native monolingual speakers of American English, had typically developing reading skills or reading disability, a nonverbal intelligence score greater than or equal to 75 (Weschler Abbreviated Scale of Intelligence-II, Perceptual Reasoning Index; Wechsler, 2011), normal or corrected-to-normal vision, and had no hearing impairment, no developmental disorders/conditions aside from learning or attention disorders, no history of seizures/epilepsy, no history of severe psychiatric disorders, and no contraindication for MRI (i.e. no ferromagnetic material in the body, claustrophobia). In total, 72 children (30 females; 42 males) participated in the study. Participants completed behavioral assessments, EEG, and fMRI across three sessions (fMRI one session, EEG one session, and behavioral one session). The EEG and fMRI sessions were conducted in counterbalanced, with a mean interval of 12 days between sessions. The mean interval between fMRI/EEG and behavioral assessments were 18 days. The final sample sizes varied across analyses due to differences in data quality and completeness: fMRI analyses (*n* = 65), EEG analyses (*n* = 66), and brain–behavior regression analyses (*n* = 56).

### 2.2 Behavioral measures

Children were asked to complete a set of standardized assessments designed to measure nonverbal intelligence, reading efficiency, reading fluency, and spelling. Nonverbal intelligence was assessed using the *Block Design (BD)* and *Matrix Reasoning (MR)* subtests of the Wechsler Abbreviated Scale of Intelligence (WASI; Wechsler, 1999). Reading efficiency was evaluated with the *Test of Word Reading Efficiency, Second Edition* (TOWRE-2; Torgesen et al., 2012). In the *Sight Word Efficiency* (SWE) subtest, children were asked to read as many real words as possible within 45 s, while in the *Phonemic Decoding Efficiency* (PDE) subtest, they read pronounceable nonwords under the same timed conditions. Children’s reading fluency and spelling skills were assessed using the *Reading Fluency* and *Spelling* subtests from the *Woodcock–Johnson III Tests of Achievement* (Schrank, 2011). The Reading Fluency subtest measures how quickly and accurately children can read simple sentences and make semantic judgments within 3 min. The Spelling subtest measures a child’s ability to correctly write words that are orally presented. The test was discontinued after six consecutive incorrect responses. Both raw and age-standardized scores were obtained for each subtest.

### 2.3 fMRI experimental design

#### 2.3.1 Single-letter detection task

The single-letter detection task comprised 321 trials distributed across four conditions: visual, auditory, audiovisual congruent (AV_congruent), and audiovisual incongruent (AV_incongruent). In the auditory-only condition, participants heard 68 spoken letter names (e.g., “*f*”) without any visual input. In the visual-only condition, 68 printed single letters (e.g., *a*) were displayed on the screen without accompanying sounds. The AV_congruent condition included 68 trials in which participants simultaneously heard and saw the same letter (e.g., hearing “*p*” while seeing *p*), whereas the AV_incongruent condition included 68 trials in which the heard and seen letters differed (e.g., hearing “*c*” while seeing *d*). To ensure participants’ attention, 49 catch trials were included, during which participants were instructed to press a button when they saw a specific letter (e.g., “*n*”). The whole task took around 20 min.

#### 2.3.2 Multi-letter detection task

The multi-letter (consonant–vowel–consonant, CVC) detection task also included a total of 321 trials across the above mentioned four conditions. In the auditory-only condition, participants completed 68 trials in which they heard spoken nonwords (e.g., “*fab*”) without any visual input. In the visual-only condition, another 68 trials presented nonwords as printed text on the screen (e.g., *bab*) without accompanying sounds. The AV_congruent condition included 68 trials in which participants simultaneously heard and saw the same nonword (e.g., hearing “*mog*” while reading *mog*), whereas the AV_incongruent condition included 68 trials in which the heard and seen nonwords differed (e.g., hearing “*rud*” while seeing *wap*). To ensure participants’ attention, 49 catch trials were included, during which participants were instructed to press a button when they saw a specific multi-letter (e.g., “*clo*”). The whole task took around 20 min.

### 2.4 EEG experimental design

#### 2.4.1 Single-letter detection task

This task followed the same experimental design as the fMRI letter detection task, except that the AV incongruent condition was not included. There were 155 visual trials, 160 auditory trials, and 152 AV_congruent trials, and 115 catch trials.

#### 2.4.2 Multi-letter detection task

This task followed the same experimental design as the fMRI nonword detection task, except that the AV incongruent condition was not included. The number of trials were the same as the single-letter detection task. See Supplementary materials S1 for more details.

### 2.5 fMRI acquisition and preprocessing

MRI data were acquired on Siemens Magnetom Prisma 3 T systems with 64-channel head coils at both Yale Magnetic Resonance Research Center (MRRC) and the University of Connecticut Brain Imaging Research Center. Functional imaging used T2*-weighted echo planar imaging (EPI) sequences (TR = 2000 ms, TE = 25–30 ms, FoV = 220 mm, flip angle = 62–90°). At Yale, voxel size was 3.4 × 3.4 × 4.0 mm; at UConn, voxel size was 2.0 × 2.0 × 2.5 mm with 52 slices and multiband acceleration factor = 2. Anatomical imaging used T1-weighted MPRAGE scans with 1.0 mm isotropic voxels (FoV = 250–256 mm, TR = 1900–2400 ms, TE = 2.15–2.52 ms, flip angle = 8–9°). Anatomical images were reconstructed and manually corrected with Freesurfer v6.0 to ensure appropriate parcellation and segmentation (Fischl, 2012). Functional images were preprocessed with fMRIPrep v1.4.1, including bias-field correction, brain extraction, normalization to the ICBM 152 nonlinear template, tissue segmentation, and motion correction procedures (Esteban et al., 2019). Normalized and extracted functional images were spatially smoothed using a 6-mm FWHM Gaussian kernel. Functional volumes with > 1 mm framewise displacement were excluded, which is a threshold previously shown to be appropriate for young children (Siegel et al., 2014). Participants who completed fewer than three out of four task runs were excluded from further analyses. In addition, participants were excluded if fewer than 70% of volumes survived motion censoring across all runs or fewer than 80% of volumes survived within the conditions of interest. In total, seven participants were excluded for not meeting these criteria.

### 2.6 EEG acquisition and preprocessing

**Fig. 1:**
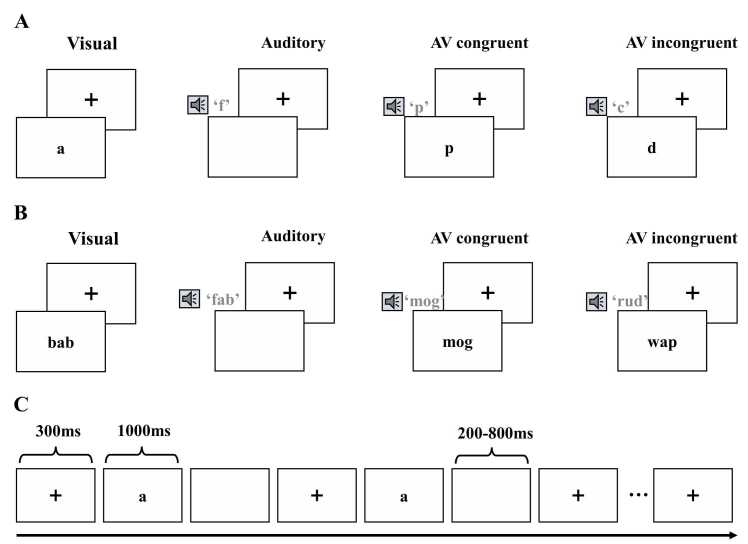
Experimental design and task paradigm for AV single-letter and multi-letter detection*.* Note. **A.** Examples of the single-letter presented in the experiments included four conditions: unimodal visual, unimodal auditory, audiovisual congruent, and audiovisual incongruent. Children were instructed to press a response button whenever a target letter was presented visually or auditorily. **B.** Examples of the multi-letter presented in the experiments across four conditions. Children were instructed to press a response button whenever a target letter was presented visually or auditorily. **C.** Illustration of the sequence and timing of stimulation trials in the target detection task. Each trial started with a fixation cross presented for 300 ms, followed by a stimulus displayed for 1000 ms. The inter-stimulus interval (ISI) varied randomly between 200 and 800 ms.

EEG was recorded using a high-density 128-electrode sensor array net placed on the scalp using Electrical Geodesics Inc. (EGI) system (MagstimEGI, Oregon, USA). The active electrodes were maintained with impedances below 10 kΩ. Raw data were sampled at 500 Hz (EGI net amps 300 system) with the Cz electrode used as reference and stored for offline analysis. The EEGLAB toolbox (Delorme and Makeig, 2004) and ERPLAB toolbox (Lopez-Calderon and Luck, 2014) were used to preprocess the EEG data. ERP and time-frequency analyses used different preprocessing pipielines. For ERP anlaysis, We re-referenced to the average of all channels, downsampled to 128 Hz and band-pass filtered the data at 0.3–30 Hz. For time-frequency analysis, data were re-referenced to the average of all channels, dowsampled to 500 Hz, and filtered with a 0.3–100 Hz band-pass and a 60 Hz notch filter. Channels with consistently poor signal quality were identified through visual inspection and statistical criteria, including excessive amplitude (exceeding ±1000 μV), abnormal kurtosis (> 3 *SD*), and spectral deviation (> 3 *SD*), and were excluded from further analysis. The rejected channels were subsequently interpolated using the average of neighboring electrodes. The EEG data were segmented into epochs of −200–800 ms and baseline-corrected using the 200 ms pre-stimulus interval. Trials were rejected if more than 20% of channels exceeded ±3 SD from the channel-wise mean amplitude computed across all time points and trials. The mean number of excluded epochs was 46.11 (*SD* = 11.64; range = 28–67), and no significant association was observed with age (*r* = −0.017, *p* = 0.872). Artifact components were automatically identified and removed based on ICLabel classification (Pion-Tonachini et al., 2019) using Independent Component Analysis (ICA) implemented in the EEGLAB toolbox. Components classified as muscle, eye, or other artifacts with category probabilities ≥ 0.9 were rejected (on average 38 components per participant). Data from six participants were excluded due to excessive channel rejection (>50%), yielding a final sample of 66 participants. Fig. 1

### 2.7 fMRI and EEG analyses

**Fig. 2:**
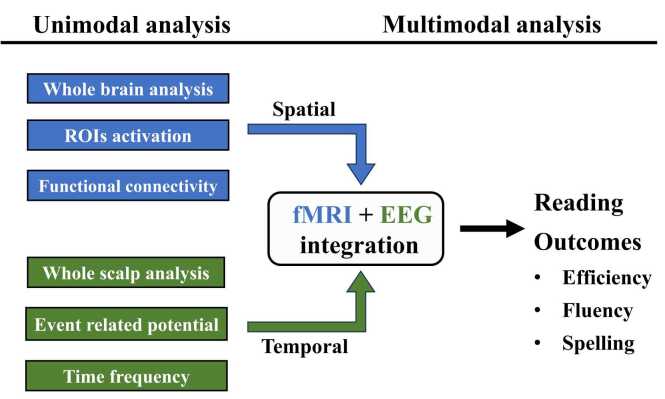
Overview of multimodal neuroimaging analyses combining fMRI and EEG. Note. fMRI analyses included whole-brain activation mapping, region-specific activation, and functional connectivity. EEG analyses focused on whole-scalp level assessments, event-related potentials (ERPs) and time–frequency dynamics. Neural measures from both modalities were examined in relation to individual differences in reading efficiency, fluency, and spelling. Both unimodal and multimodal models—integrating fMRI and EEG metrics—were compared to evaluate their respective contributions in explaining variance across reading performance measures.

Neural indices derived from both fMRI and EEG were analyzed to capture complementary spatial and temporal aspects of AV integration and their relationships with reading skills. See Fig. 2 for an overview of the multimodal fMRI–EEG analytic framework.

#### 2.7.1 fMRI whole brain analysis

For whole brain analysis, a random-effect generalized linear model (GLM) was calculated with eight predictors (single-letter_visual, single-letter_auditory, single-letter_AVCongruent, single-letter_AVIncongruent, multi-letter_visual, multi-letter_auditory, multi-letter_AVCongruent, multi-letter_AVIncongruent) and six movement parameters for each participant in the first level analysis. First-level analyses on subject level included the contrasts of each condition against baseline and the comparisons between the eight conditions (i.e., single-letter_visual, single-letter_auditory, single-letter_AVCongruent, single-letter_AVIncongruent, multi-letter_visual, multi-letter_auditory, multi-letter_AVCongruent, multi-letter_AVIncongruent).

To examine additive enhancement effects and their modulation by letter type, the first whole-brain model was specified as a 2 × 2 mixed-effects design. The fixed factors included modality (audiovisual AV vs. the mean of unimodal auditory and visual responses, (A + V)/2) and letter type (single-letter vs. multi-letter), with participant modeled as a random intercept to account for between-subject variability. This design allowed testing the main effect of modality (i.e., AV > (A + V)/2), the main effect of letter type, and their interaction. Statistical significance was assessed using a voxel-wise threshold of *p* < .001 (uncorrected), with cluster-level correction for multiple comparisons at *p* < .05 FWE. Following Beauchamp (2005), we used the mean additivity criterion (AV > (A + V)/2) rather than the stricter super-additivity criterion (AV > A + V), as the latter often fails to identify well-established multisensory regions such as the superior temporal sulcus.

To further investigate how AV congruency modulates neural responses during print–sound integration, a second whole-brain model was implemented focusing on the two AV conditions for both single-letter and multi-letter tasks. Contrast images from the first-level analyses were entered into a second-level flexible factorial design in SPM12 (Friston et al., 2008), with letter type (single-letter, multi-letter) and AV congruency (AV_congruent, AV_incongruent) specified as within-subject factors, resulting in a 2 × 2 repeated-measures design matrix. Subject was modeled as a random effect to capture individual differences in brain activation. Group-level contrasts were computed to examine the main effects of letter type and congruency, as well as their interaction. Statistical significance was determined using a voxel-wise threshold of *p* < .001 (uncorrected), with cluster-level correction for multiple comparisons at *p* < .05 FWE.

#### 2.7.2 Anatomically defined ROI-based analysis

In addition to the exploratory whole-brain analysis, confirmatory ROI analysis was also performed. Several regions known to be involved in AV integration were selected, including the STG, IFG, MTG, AG and FG in the left hemisphere (Blau et al., 2010, Karipidis et al., 2018, Kast et al., 2011, Kronschnabel et al., 2014, Plewko et al., 2018). ROIs were anatomically defined using the AAL atlas (Tzourio-Mazoyer et al., 2002, Fig. 6A). For each participant, contrast estimates (*β* values) were extracted separately from each ROI using the MarsBaR toolbox (Brett et al., 2002) in SPM12 (Friston et al., 2008). The extracted *β* values were then entered into the same factorial mixed-effects models used in the whole-brain analyses—namely, (1) a 2 × 2 design with letter type (single-letter vs. multi-letter) and AV Congruency (congruent, incongruent), and (2) a 2 × 2 design with modality (AV vs. (A+V)/2) and letter type (single-letter vs. multi-letter) with participant modeled as a random effect. Statistical significance was determined at *α* = 0.05. To control for multiple comparisons across ROIs, FDR correction was applied (*q* < 0.05).

#### 2.7.3 fMRI functional connectivity analysis

To determine whether AV integration relies on functional coupling between key cortical regions, we conducted ROI-to-ROI generalized psychophysiological interaction (gPPI) analyses using the CONN functional connectivity toolbox (Whitfield-Gabrieli and Nieto-Castañon, 2012). Analyses targeted ROI pairs identified the activation results from the current study. For the additive enhancement model, ROIs included the left and right STG (posterior divisions). For the congruency model, two anatomically defined ROIs were selected—the left inferior frontal gyrus (IFG; pars triangularis and pars orbitalis) and the left superior temporal gyrus (STG; posterior division), see Supplementary materials eFigure 3. For each participant, denoised BOLD time series were extracted from each ROI defined in the AAL atlas (Tzourio-Mazoyer et al., 2002). The CONN toolbox implemented gPPI models that included (i) the physiological time series for each ROI, (ii) psychological regressors for each condition, (iii) their interaction terms, and (iv) six motion parameters as nuisance covariates. Functional coupling was computed for the predefined ROI pairs. Specifically, we tested whether (a) connectivity between the left and right STG was stronger in AV compared to (A+V)/2, and (b) connectivity between the left IFG and left STG increased during AV_incongruent than AV_congruent trials. At the group level, one-sample *t*-tests were performed on individual gPPI beta estimates to identify consistent connectivity effects across participant.

#### 2.7.4 EEG whole scalp analysis

For the whole scalp analysis, data from all 128 scalp electrodes were included. Time was locked to the stimulus, and the test was performed on all time points between 0 and 700 ms (i.e., 11648 total comparisons). The baseline interval was the 200 ms preceding stimulus onset. Repeated-measures analyses of variance (ANOVAs) were conducted on event-related potentials (ERPs) using the FieldTrip Mass Univariate Toolbox (FMUT; Fields and Kuperberg, 2020), which extends the framework of the Mass Univariate ERP Toolbox (Groppe et al., 2011). We tested the effects of letter type (single-letter vs. multi-letter), and modality (AV vs. (A+V)/2) on the ERP data, resulting in a 2 × 2 within-participants design.

Mass univariate repeated-measures ANOVAs were performed at each electrode and time point using the FMUT function FfdrGND. To correct for multiple comparisons, we employed the Benjamini–Hochberg false discovery rate (FDR) procedure (Benjamini and Hochberg, 1995) with *q* = 0.05. FDR controls the expected proportion of false positives, offering greater sensitivity to short-lived or spatially focal effects compared to family-wise error rate or cluster-based permutation approaches (Maris and Oostenveld, 2007). For each comparison, observed F-values were calculated and evaluated against critical thresholds derived from the adjustment. Significant effects were visualized with raster plots, providing a spatiotemporal map of reliable ERP differences across the scalp.

#### 2.7.5 ERP analysis

The electrodes used for the ERP analyses were selected a priori based on two independent criteria: (1) cortical regions implicated by our fMRI results and (2) previous meta- analytical review on audiovisual and reading-related processing (Gao et al., 2023). This resulted in a left frontotemporal region of interest that encompassed all left frontal and temporal scalp sites, which was defined independently of, and more broadly than, the clusters identified in the EEG whole-scalp statistical maps. Grand-averaged ERP waveforms were then extracted for both the AV and (A + V)/2 conditions, with data combined across the two tasks. These waveforms were used to identify the ERP components, and the mean amplitude differences between the AV and (A + V)/2 conditions were subsequently computed and statistically tested to quantify the multisensory integration effects.

#### 2.7.6 EEG time frequency analysis

For frequency-domain preprocessing, EEG data were processed using the EEGLAB toolbox (Delorme and Makeig, 2004) and the ERPLAB toolbox (Lopez-Calderon and Luck, 2014). Channels with consistently poor signal quality were identified and rejected through visual inspection and statistical criteria (excessive amplitude: ±1000 μV; abnormal kurtosis: > 3 SD; spectral deviation: > 3 SD). Rejected channels were then interpolated using the average of neighboring electrodes. The data were segmented into epochs ranging from –600–800 ms relative to stimulus onset. Trials were rejected if more than 20% of channels exceeded ±3 SD from the channel-wise mean amplitude computed across all time points and trials. Artifacts were automatically removed using ICA implemented in the EEGLAB toolbox.

Single-trial EEG data were transformed into the time–frequency domain using wavelet analysis (EEGLAB function newtimef.m). Output frequencies ranged from 4 to 80 Hz in 1-Hz steps. The wavelet width scaled with frequency, from 3 cycles at 4 Hz to 25 cycles at 80 Hz, providing an optimal trade-off between frequency and time resolution, particularly important for analyzing low-frequency pre-stimulus activity. The baseline period was defined as the interval from –200–0 ms, immediately preceding stimulus onset.

To complement the whole-scalp and ERP findings, we extracted the significant ROI channels and time windows identified in those analyses. We then computed frequency-band results (4–80 Hz) separately for the AV and (A + V)/2 conditions. Time–frequency representations were computed and visualized from 0 to 300 ms for both conditions, as well as for their difference (AV – (A + V)/2). Data from the two tasks were merged, and statistical tests were conducted to examine significant differences in oscillatory power between the AV and (A + V)/2 conditions across distinct frequency bands.

### 2.8 Brain–behavior regression analyses

To examine how neural markers of AV integration relate to individual differences in reading skills, we conducted a series of multiple linear regression models, each testing the association between one neural index and one behavioral outcome. For fMRI, additive enhancement effects were observed in the left IFG and bilateral STG, and congruency effects emerged in the left IFG and left STG. Beta estimates from these anatomically defined ROIs were extracted for each participant and entered as predictors in separate regression models. Each model included one neural index (ROI activation or connectivity) and one reading outcome (reading efficiency, reading fluency, or spelling), with age and nonverbal intelligence (WASI Performance IQ) included as covariates. Parallel regression analyses were conducted for EEG indices.

In addition to regional activation, we examined functional connectivity derived from the gPPI analyses. Specifically, connectivity strength between the left IFG and left STG from the congruency contrast was tested as a predictor of reading performance. Parallel multiple linear regression analyses were conducted for EEG-based neural indices, including the P200 amplitude difference and theta-band (4–7 Hz) power difference between the AV and (A + V)/2 conditions. All models included age and nonverbal intelligence as covariates. Analyses were implemented in R 4.1.0 (R Core Team, 2021) using base linear modeling functions.

### 2.9 Unimodal and multimodal neural predictors of reading

To further evaluate the unique and combined contributions of neural measures to reading performance, we constructed separate unimodal models for fMRI and EEG data, as well as an integrated multimodal model that combined predictors from both modalities. All models included age and nonverbal intelligence as covariates. The fMRI model tested for additive enhancement effects in the left STG, congruency effects in the left IFG and left STG, and functional connectivity between the left IFG and left STG derived from the congruency model. The EEG model tested for P200 amplitude and theta-band power over left frontotemporal electrodes. The multimodal model combined all fMRI- and EEG-based predictors within a single regression framework to test whether integrating spatial and temporal neural indices of AV integration improved prediction of reading skills beyond either modality alone. Model fit was quantified using *R²*, and improvements in explained variance were evaluated using nested F-tests.

## 3 Results

### 3.1 Behavioral

**Fig. 3:**
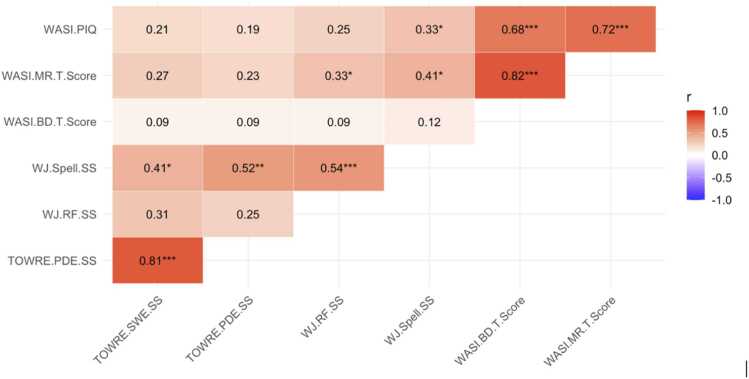
Pearson partial correlations among behavioral measures (controlling for age)*.* Note. TOWRE.SWE.SS = Test of Word Reading Efficiency – Sight Word Efficiency Standardized Score; TOWRE.PDE.SS = Test of Word Reading Efficiency – Phonemic Decoding Efficiency Standardized Score; WJ.RF.SS = Woodcock-Johnson Reading Fluency Standardized Score; WJ.Spell.SS = Woodcock-Johnson Spelling Standardized Score; WASI.BD.T.Score = Wechsler Abbreviated Scale of Intelligence – Block Design T-score; WASI.MR.T.Score = Wechsler Abbreviated Scale of Intelligence – Matrix Reasoning T-score; WASI.PIQ = Wechsler Abbreviated Scale of Intelligence – Performance IQ.**.** Asterisks indicate significance levels: * indicates *p* < 0.05; ** indicates *p* < 0.01; *** indicates *p* < 0.001.

Of the children who underwent both fMRI and EEG scanning, 56 (22 females, 34 males; *M* age = 8.90 years, *SD* = 1.02) completed all behavioral assessments. As shown in Table 1, the sample displayed a broad range of cognitive and reading abilities. Participants demonstrated generally average nonverbal reasoning ability, with a mean WASI Performance IQ of 94.68 (SD = 15.51, range = 75–139), indicating variability across individuals. Reading-related measures showed particularly pronounced individual differences. Standardized scores ranged from 48 to 118 for TOWRE SWE, 48–117 for TOWRE PDE, 70–139 for Reading Fluency, and 63–137 for Spelling, reflecting wide variation in reading efficiency, fluency, and spelling across participants. Using the conventional cutoff of SS < 85 (1 SD below the normative mean) on TOWRE-2 word reading measures, 17 participants scored below this threshold on SWE, 16 participants on PDE, and 19 participants on at least one subtest. Five children had estimated IQ scores between 75–84. The mean Performance IQ was 94.68 (SD = 15.51), indicating that the majority of participants fell within the average range. Four children were diagnosed with attention disorders. The two TOWRE subtests—SWE and PDE are highly correlated (*r* = .81, *p* < .001, Fig. 3). Both TOWRE measures also showed moderate associations with spelling (WJ.Spell.SS; *r* = .41–.52, Fig. 3). Correlations with nonverbal reasoning measures (WASI.PIQ, WASI.MR.T.Score, WASI.BD.T.Score) were smaller and mostly nonsignificant (Fig. 3).

**Table 1:** Descriptive statistics for behavioral measures (N = 56).

**Measure**	**Mean (SD)**	**Range**
**WASI**		
Block Design (T)	42.56 (15.54)	21–68
Matrix Reasoning (T)	43.00 (14.53)	19–80
Performance IQ	94.68 (15.51)	75–139
**TOWRE-2**		
SWE Raw	45.51 (17.92)	2–85
SWE SS	90.68 (21.18)	48–118
PDE Raw	18.13 (10.69)	1–47
PDE SS	88.79 (18.54)	48–117
**Woodcock–Johnson IV**		
Reading Fluency Raw	31.74 (13.15)	6–62
Reading Fluency SS	101.09 (13.14)	70–139
Spelling Raw	26.31 (8.48)	13–51
Spelling SS	97.83 (18.07)	63–137

*Note.* WASI = Wechsler Abbreviated Scale of Intelligence; TOWRE-2 = Test of Word Reading Efficiency, Second Edition; SWE = Sight Word Efficiency; PDE = Phonemic Decoding Efficiency; SS = Standard Score.

### 3.2 fMRI whole brain results

#### 3.2.1 Additive enhancement model

**Fig. 4:**
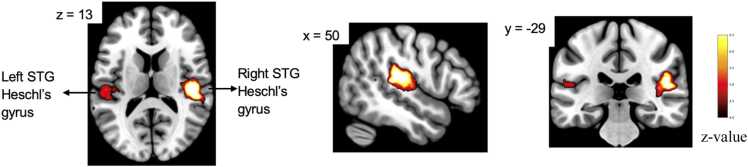
Whole brain activation map from additive enhancement model**.** Note. Brain regions showing significantly greater activation for audiovisual compared to the mean of the unimodal auditory and visual conditions (AV > (A+V)/2). Results are displayed on the MNI152 template.Significant clusters were observed in bilateral superior temporal gyrus and bilateral Heschl’s gyrus. Threshold: voxelwise *p* < 0.001, cluster-level FWE corrected *p* < 0.05. Color bar indicates *z*-values.

For the fMRI additive enhancement model, a main effect of modality was observed, with AV stimuli eliciting significantly greater activation than the mean of the unisensory conditions (A+V)/2 in bilateral superior temporal gyrus (left STG: *β =* 0.51*, SE =* 0.17*, t* = 3.02*, p-*FWE *=* 0.021; right STG: *β =* 0.47*, SE =* 0.16*, t* = 2.88*, p-*FWE *=* 0.027) and in bilateral Heschl’s gyrus (left HG: *β* = 0.44*, SE* = 0.15*, t* = 2.91*, p-* FWE = 0.025; right HG: *β* = 0.42*, SE* = 0.14*, t* = 2.86*, p-*FWE = 0.029), see Fig. 4. No other main and interaction effects survived for whole brain analysis.

#### 3.2.2 Congruency model

**Fig. 5:**
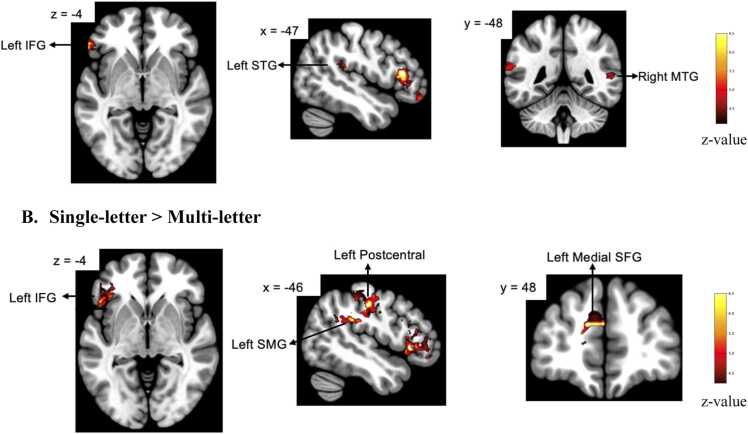
Whole brain activation map from congruency model**.** Note. **A.** Brain regions showing significantly greater activation for incongruent compared to congruent audiovisual multi-letter trials (AV_Incongruent > AV_Congruent). Significant clusters were observed in the left inferior frontal gyrus (triangularis, orbitalis), left superior temporal gyrus, and right middle temporal gyrus. **A.** Brain regions showing significantly greater activation for single-letter compared to multi-letter (single-letter > multi-letter). Significant clusters were observed in the left inferior frontal gyrus (triangularis, orbitalis), postcentral gyrus, and supramarginal gyrus. Threshold: voxelwise *p* < 0.001, cluster-level FWE corrected *p* < 0.05. Color bar indicates z-values.

The interaction between AV congruency and letter type was significant (*β* = −0.21, *SE* = 0.39, *p*-FWE = 0.013). For the multi-letter condition, a significant congruency effect was observed, with AV incongruent trials eliciting greater activation than AV congruent trials in several regions. These included the left inferior frontal gyrus, triangularis (*β* = −0.41*, SE* = 0.15*, t* = -2.74*, p-*FWE = 0.032), the left inferior frontal gyrus, orbitalis (*β* = −0.46*, SE* = 0.16*, t* = -2.91*, p-*FWE = 0.018), the left superior temporal gyrus (*β* = −0.44*, SE* = 0.15*, t* = -2.87*, p-*FWE = 0.023), and the right middle temporal gyrus (*β* = −0.39*, SE* = 0.14*, t* = -2.69*, p*-FWE = 0.036), see Fig. 5A. There was no congruency effect for single-letter condition (*p* > 0.1). The main effect of letter type was observed, with letter stimuli eliciting significantly greater activation than multi-letter in several left-hemisphere regions. This included the inferior frontal gyrus, triangularis (*β* = 0.42*, SE* = 0.15*, t* = 2.81*, p*-FWE = 0.034), the inferior frontal gyrus, orbitalis (*β* = 0.48*, SE* = 0.14*, t* = 3.29*, p*-FWE = 0.012), the postcentral gyrus (*β* = 0.39*, SE* = 0.13*, t* = 2.95*, p*-FWE = 0.018), and the supramarginal gyrus (*β* = 0.36*, SE* = 0.14*, t* = 2.63*, p-*FWE = 0.040), see Fig. 5B. A marginal effect was also observed in the superior medial frontal gyrus (*β* = 0.28*, SE* = 0.16*, t* = 1.75*, p-*FWE = 0.097).

Table 2 provided a summary for significant brain regions identified in whole-brain analyses of additive enhancement and congruency models.

**Table 2:** Significant brain regions identified in whole-brain analyses of additive enhancement and congruency models.

**Brain Area**	**Hemisphere**	**X**	**Y**	**Z**	**Voxels**	***p*****-FWE**
**Additive enhancement model**						
AV > (A+V)/2						
Heschl	Left	-43	19	11	89	0.041
Heschl	Right	57	-22	7	87	0.025
Temporal_Sup	Left	-62	-26	8	123	0.037
Temporal_Sup	Right	47	14	-17	146	0.029
**Congruency model**						
Multi-letter: AV_incongruent> AV_congruent						
Frontal_Inf_Tri	Left	-44	30	10	102	0.032
Frontal_Inf_Orb	Left	-42	26	-8	45	0.018
Temporal_Sup	Left	-62	-26	8	44	0.023
Temporal_Mid	Right	56	-28	2	50	0.036
Single-letter > multi-letter						
Frontal_Inf_Tri	Left	-44	20	10	112	0.034
Frontal_Inf_Orb	Left	-42	26	-8	99	0.012
Frontal_Sup_Medial	Left	-8	52	24	60	0.097
PostCentral	Left	-40	-30	52	212	0.018
SupraMarginal	Left	-60	-40	40	131	0.040

*Note.* AV = audiovisual; A = auditory; V = visual; *p*-FWE = *p*-value corrected for family-wise error. Brain regions: Frontal_Inf_Tri = inferior frontal gyrus, pars triangularis; Frontal_Inf_Orb = inferior frontal gyrus, pars orbitalis; Temporal_Sup = superior temporal gyrus; Temporal_Mid = middle temporal gyrus; Frontal_Sup_Medial = superior medial frontal gyrus. Coordinates (X, Y, Z) are in MNI space.

### 3.3 Anatomically defined ROI analysis results

**Fig. 6:**
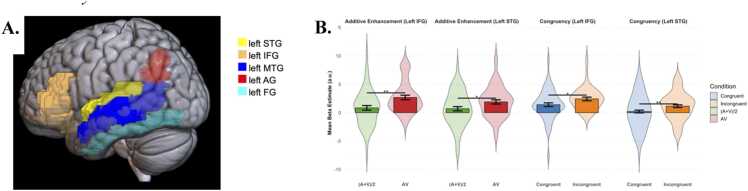
ROI analysis results. Note. **A.** Anatomically defined AAL ROIs, including left STG, left IFG, left MTG, left AG and left FG. **B.** ROI activation in the additive enhancement and congruency models.

In the additive enhancement model, there was a significant main effect of modality, with higher activation for AV than for the mean of unimodal auditory and visual inputs ((A + V)/2 (*β* = 1.12, *SE* = 0.41, *p* = 0.017). In the congruency model, there was a main effect of congruency, with greater activation for incongruent than congruent conditions (*β* = 0.95, *SE* = 0.32, *p* = 0.004). Simple effects analyses further revealed that both the left IFG and left STG showed stronger responses to AV than (A + V)/2 stimuli (*t* = 3.23, *p* = 0.002; *t* = 2.29, *p* = 0.025, respectively). Simple effects analyses also revealed that both the left IFG and left STG showed stronger responses to incongruent than congruent AV pairs (*t* = 2.21, *p* = 0.031; *t* = 2.73, *p* = 0.008, respectively, Fig. 6B).

### 3.4 Functional connectivity analysis

For the additive enhancement contrast (AV > (A+V)/2), connectivity between the left and right STG (posterior division and Heschl’s gyrus) was not significant (*p* = 0.392). For the congruency contrast (AV incongruent > AV congruent), connectivity between the left IFG (pars triangularis and pars orbitalis) and the left STG (posterior division) was significantly stronger in incongruent than congruent AV trials (*t* = 3.42, *p* < .001).

### 3.5 EEG whole-scalp results

**Fig. 7:**
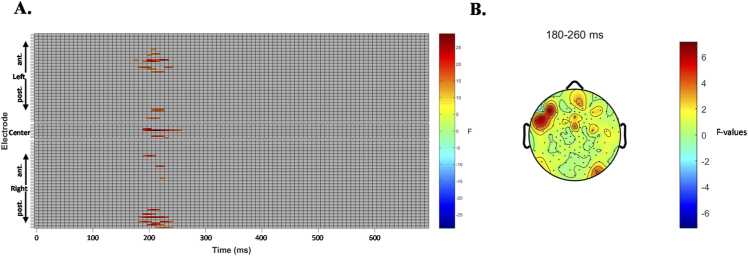
Stimulus-locked AV integration effects [(AV) − (A+V)/2]. Note. **A.** Results of the whole-scalp analysis showing the main effect of modality (AV vs. (A+V)/2), collapsed across stimulus types. Significant differences were found primarily at left anterior electrodes (e.g., E6, E7, E28, E29, E65), as well as other central and posterior sites (e.g., E83, E90, E74), starting around 179 ms and ending around 258 ms. **B.** Topographic Map of F-values. Topographical maps of the modality effects within the 0–700 ms time window.

**Fig. 8:**
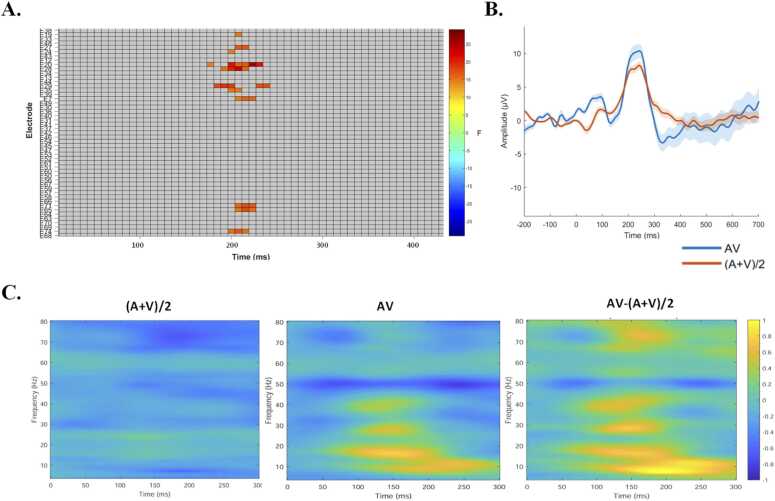
EEG indices of AV integration in the left frontotemporal regions. Note. **A.** Whole-scalp analysis of modality effects in the left hemisphere. Significant differences between AV and (A+V)/2 were observed in the frontal and temporal lobes. **B.** Hypothesis-driven ROI ERP analysis. Grand-averaged waveforms for the AV and (A+V)/2 conditions were shown for electrodes over the whole left frontal–temporal regions (E6, E7, E11, E12, E13, E18, E19, E20, E21, E22, E23, E24, E25, E26, E27, E28, E29, E30, E32, E33, E34, E35, E36, E38, E39, E43, E44, E48, E128). **C.** Grand-average neural power for AV, (A+V)/2 conditions and their difference in the left frontal and temporal lobes. Time 0 ms corresponded to the stimulus onset.

In the whole-scalp EEG analysis, both stimulus type (single-letter vs. multi-letter) and multisensory integration effects (AV vs. (A+V)/2) were examined using mass-univariate repeated-measures ANOVAs with a 2 × 2 within-participant design. Specifically, whole-scalp analysis of the 700-ms interval following stimulus onset revealed a significant main effect of modality. In contrast, no significant main effects of letter type or interaction effects were observed. Therefore, Fig. 7 presented the main effect of modality (AV vs. (A+V)/2), collapsed across stimulus types. Significant differences were primarily observed over left anterior electrodes (e.g., E6, E7, E28, E29, E65), as well as at several central and posterior sites (e.g., E83, E90, E74) (Fig. 7A). Modality effects (AV > (A+V)/2) emerged early, beginning around 179 ms after stimulus onset and persisting until approximately 258 ms, with peak differentiation occurring between 203 and 211 ms across multiple frontal and central electrodes. Topographic maps revealed a widespread modality effect with a clear left anterior predominance (Fig. 7B). Fig. 8A illustrated the specific channels located over the left frontotemporal region, which, consistent with the MRI findings, emerged as the primary locus of audiovisual integration, showing significant activity between 190 and 230 ms.

### 3.6 ERP results

We plotted the averaged waveforms over the left frontotemporal electrodes (specific channels see the Supplementary materials eFigure1) and observed a clear P200 component. Further analysis revealed that the AV condition elicited significantly larger amplitudes than the mean of the unimodal conditions (A + V)/2, in left frontotemporal region (*t* = 2.03, *p* = 0.046; Fig. 8B). No significant differences were observed between the two tasks (eFigure 2).

### 3.7 EEG time-frequency results

We next conducted time–frequency analysis over the left frontotemporal region within the 190–230 ms time window to further elucidate the neural mechanisms underlying AV integration and complement the ERP findings. Modality effects were tested across frequency bands (theta: 4–7 Hz; beta: 13–30 Hz; gamma: 30–80 Hz). A significant modality effect was observed from 190 to 230 ms (*t* = 2.56, *p* = 0.016), characterized by greater theta-band (4–7 Hz) power in the AV condition compared to the (A+V)/2 condition (Fig. 8C). No other significant modality-related effects were found across frequency bands (*ps* >.05).

### 3.8 Brain-behavior results

**Fig. 9:**
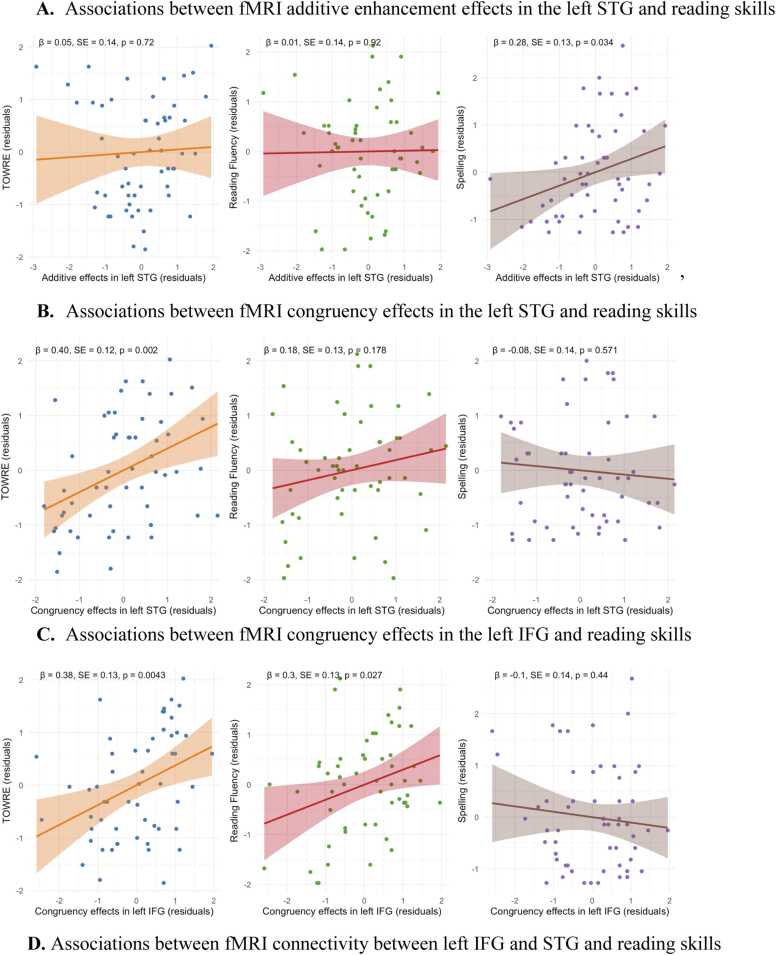

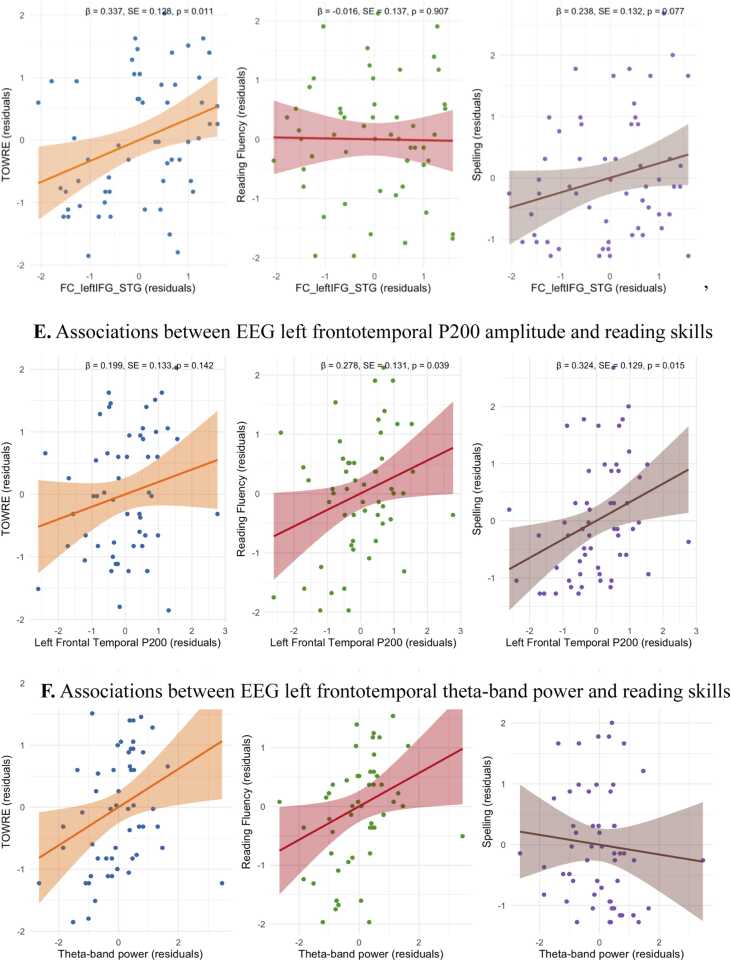
Association between neural indices and reading skills**.** Note. Scatterplots showing associations between neural indices and reading measures. STG = superior temporal gyrus; IFG = inferior frontal gyrus; TOWRE = Test of Word Reading Efficiency. Shaded regions represent 95% confidence intervals.

For fMRI, results showed that the additive enhancement effect (AV > (A+V)/2) in left STG was positively associated with spelling (*β* = 0.28, *SE* = 0.13, *p* = .034) but not with TOWRE (*p* = 0.72) or reading fluency (*p* = 0.92), Fig. 9A. Additive enhancement effects in left IFG and right STG were not associated with any reading skills (*p* > 0.1). The congruency effect in left STG was positively associated with TOWRE (*β* = 0.40, *SE* = 0.12, *p* = 0.002) but not with reading fluency (*p* = 0.18) and spelling (*p* = 0.57), Fig. 9B. The congruency effect in left IFG was positively associated with TOWRE (*β* = 0.38, *SE* = 0.13, *p* = 0.004) and reading fluency (*β* = 0.30, *SE* = 0.13, *p* = 0.027) but not with spelling (*p* = 0.44), Fig. 9 C. A significant positive relationship was observed between left IFG–STG connectivity and TOWRE (*β* = 0.337, *SE* = 0.128, *p* = 0.011), a marginally significant relationship with spelling (*β* = 0.238, *SE* = 0.132, *p* = 0.077), but not with reading fluency (*p* = 0.907), Fig. 9D. For EEG, results showed that the difference in mean P200 amplitude between the additive enhancement effect (AV- (A+V)/2) was positively associated with reading fluency (*β* = 0.28, *SE* = 0.13, *p* = 0.039) and spelling (*β* = 0.32, *SE* = 0.13, *p* = 0.015), but not with TOWRE (*p* = 0.142), Fig. 9E. Greater theta power in the additive enhancement effect was positively associated with TOWRE scores (*β* = 0.31, *SE* = 0.13, *p* = 0.021) and reading fluency (*β* = 0.28, *SE* = 0.13, *p* = 0.034), but not with spelling (*p* = 0.555), Fig. 9F.

### 3.9 Unimodal vs. multimodal neural predictors of reading

To evaluate the unique and combined contributions of neural measures to reading performance, we constructed separate unimodal models for fMRI and EEG, as well as an integrated multimodal model. Each model included age and nonverbal intelligence as covariates. For the fMRI model, predictors included additive effects in the left STG, congruency effects in the left STG and IFG, and functional connectivity between the left IFG and STG. For the EEG model, predictors included the P200 amplitude and theta-band power. The multimodal model combined all predictors from both modalities to assess whether integrating spatial and temporal neural indices of AV integration improved the prediction of reading skills. To correct for multiple comparisons, we employed the Benjamini–Hochberg false discovery rate (FDR) procedure (Benjamini and Hochberg, 1995).

**Fig. 10:**
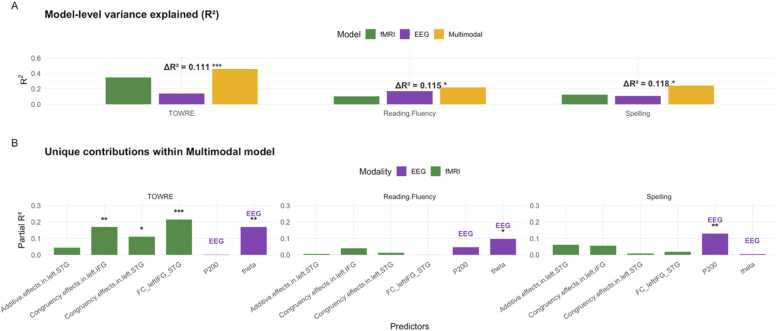
Comparison of fMRI, EEG, and multimodal neural indices in predicting reading skills. Note. A. Model-level variance was explained (*R²*) by fMRI-only, EEG-only, and multimodal (fMRI + EEG) models for each reading skill. Δ*R²* indicated the increase in explained variance when EEG predictors were added to the fMRI model. B. Unique contributions of each predictor within the multimodal model. Bars represented partial *R²* values for fMRI (green) and EEG (purple) predictors. Asterisks denote significance levels (*p* < 0.05 *, *p* < 0.01 **, *p* < 0.001 ***). TOWRE = Test of Word Reading Efficiency; fMRI = functional magnetic resonance imaging; EEG = electroencephalography.

The multimodal model significantly improved model fit in TOWRE performance (*fMRI* R² = 0.347, *Multimodal* R² = 0.457, ΔR² = 0.111, *F*(2, 49) = 4.99, *p* = 0.0106), Fig. 10A. In the multimodal model, greater functional connectivity between the left IFG and STG predicted higher TOWRE scores (*β* = 6.89, *SE* = 1.88, *p* = 0.0006). Stronger congruency effects in the left IFG (*β* = 4.51, *SE* = 1.43, *p* = 0.0028) and in the left STG (*β* = 3.01, *SE* = 1.22, *p* = 0.017) were significant predictors. Larger theta activity was also associated with better performance (*β* = 3.28, *SE* = 1.04, *p* = 0.0027). Additive effects in the left STG (*p* = 0.14) and P200 amplitude (*p* = 0.82) were not significant. For reading fluency, the multimodal model significantly improved model fit (*fMRI* R² = 0.101, *Multimodal* R² = 0.216, ΔR² = 0.115, *F*(2, 47) = 3.45, *p* = 0.0399), Fig. 10A. Only one predictor reached significance: larger theta activity predicted better fluency performance (*β* = 2.18, *SE* = 0.98, *p* = 0.030). Other predictors were not significant (*ps* > 0.1). The multimodal model significantly improved model fit for spelling (*fMRI* R² = 0.124, *Multimodal* R² = 0.242, ΔR² = 0.118, *F*(2, 49) = 3.8, *p* = 0.0291), Fig. 10B. The only significant predictor was the EEG P200 amplitude (*β* = 0.48, *SE* = 0.18, *p* = 0.009). Other predictors did not reach significance (*ps* > 0.1). See Supplementary material S2 for more analyses.

## 4 Discussion

The present study aimed to investigate the neural mechanisms supporting AV integration during reading-related processing and to examine how these neural patterns relate to individual differences in reading skills. Using multimodal neuroimaging methods, including fMRI and EEG, we characterized neural responses across both spatial and temporal dimensions—capturing whole-brain activation and functional connectivity as well as transient ERP and time–frequency dynamics. Our findings revealed robust AV integration effects in both modalities, with significant activation differences in left-lateralized frontotemporal networks, particularly the left IFG and STG. Complementary EEG analyses revealed corresponding effects within the 190–230 ms window, including enhanced ERP responses and increased theta-band power in the left frontotemporal regions, reflecting early integration of print and speech information. Furthermore, distinct neural measures contributed uniquely to reading performance: fMRI activation within the left IFG and STG, functional connectivity between these regions, as well as the amplitude of early P200 components and task-related theta power, were each associated with different aspects of reading skills. Finally, multimodal models combining fMRI and EEG measures explained more variance in reading skills than unimodal models.

### 4.1 Neural dynamics of AV integration in the reading brain

A central finding of this study was the presence of two complementary markers of AV integration: additive enhancement and congruency effects. Additive enhancement was observed in fMRI, where AV stimuli elicited stronger activation than the mean of unimodal auditory and visual inputs. Significant activation was found in the bilateral STG and Heschl’s gyrus, consistent with previous evidence that auditory association cortices act as convergence hubs for multisensory binding (Beauchamp, 2005, Calvert, 2001, van Atteveldt et al., 2004). These additive enhancement effects are considered signatures of low-level integration, reflecting the automatic convergence of redundant auditory and visual inputs. In contrast, congruency effects reflect a higher-order mechanism through which the brain differentiates consistent (print–speech sound matches) from inconsistent pairings. Our results showed that incongruent pairings elicited stronger activation than congruent ones, but only for multi-letter condition, with significant effects in the left IFG and left STG, regions central to phonological processing and conflict monitoring (Kronschnabel et al., 2014, Richlan, 2019). This pattern is consistent with Wang et al. (2020), who reported similar incongruency effects for multi-letter in German-speaking children of comparable age, suggesting that increased engagement of the IFG and STG during nonword processing reflects a general mechanism of phonological conflict resolution across orthographies. Our findings on the increased functional connectivity between the left IFG and STG further complemented this result, indicating that successful resolution of print–speech mismatches depend not only on local activation within these regions but also on their effective coordination as a frontotemporal network supporting controlled phonological integration.

However, unlike classic findings showing stronger activation for congruent than incongruent letter–sound pairs in typical readers (van Atteveldt et al., 2004, 2007), our results did not reveal a congruency advantage for single-letter. One possible explanation is that for highly familiar letter–sound mappings, integration may occur automatically and efficiently, producing minimal differential activation between congruent and incongruent conditions.

EEG provided complementary temporal information on the dynamics of AV integration. Significant AV-related modulation was observed in the P200 window (approximately 190–230 ms) over left frontotemporal electrodes. This P200 effect reflects the rapid recruitment of frontal resources to facilitate phonological classification, consistent with prior studies linking the P200 to attentional allocation and grapheme–phoneme binding (Liu et al., 2013, Hocking and Price, 2009). Importantly, the timing of the P200 effect corresponds to the fMRI evidence of activation in left frontotemporal regions, indicating that comparable neural networks may contribute to early, automatic phonological binding. The early onset of this effect indicates that top–down coordination between left temporal and frontal areas could occur within a few hundred milliseconds after stimulus presentation, supporting fast and efficient mapping between print and sound. Complementary to the transient P200 response, time–frequency analysis revealed increased theta-band (4–7 Hz) activity for AV compared to unisensory conditions over frontotemporal regions. Theta oscillations have been linked to phonological working memory, top–down control, and cross-modal integration (Bastiaansen and Hagoort, 2006, Hagoort, 2014), suggesting that this enhancement reflects sustained coordination between auditory and visual systems during print–speech mapping. In combination, the oscillatory and ERP findings indicate that AV integration engages both rapid, transient neural responses and prolonged rhythmic synchronization within the frontotemporal network, supporting efficient coupling between perceptual and phonological processes.

Taken together, these multimodal findings indicate that additive enhancement in the STG and Heschl’s gyrus supports low-level sensory convergence, whereas congruency effects in the STG and IFG reflect higher-order phonological conflict monitoring and evaluation of cross-modal consistency. Functional connectivity analyses further revealed that stronger coupling between the left IFG and STG was associated with better reading performance, suggesting that coordination between auditory and language-related regions facilitates efficient processing of AV information. At the electrophysiological level, the P200 component reflects rapid phonological binding processes, while time–frequency analyses revealed increased theta-band synchronization within the left frontotemporal network, indexing early integration of print and speech. The convergence of spatial (fMRI) and temporal (EEG) findings underscores the value of a multimodal approach for characterizing how sensory convergence, oscillatory coordination, and cognitive control jointly support efficient AV integration during reading.

### 4.2 Neural correlates of individual differences in reading

The present study examined how individual variability in neural measures of AV integration related to reading skills. A systematic pattern emerged in which different neural markers were selectively associated with distinct aspects of reading skill, underscoring the multidimensional nature of the network supporting print-speech mapping.

At the perceptual level, the additive enhancement in the left STG was positively associated with spelling performance, consistent with the idea that stronger automatic convergence of auditory and visual inputs supports the development of stable orthographic representations. At a higher level of processing, congruency effects in the left STG were positively related to TOWRE performance, suggesting that sensitivity to the consistency of print–speech pairings facilitate more efficient decoding. Although both findings implicate STG, the underlying mechanisms differ: additive enhancement reflects low-level sensory integration, whereas the congruency effect captures higher-order phonological mapping efficiency. In parallel, congruency effects in the left IFG were positively associated with both TOWRE and reading fluency, consistent with the IFG’s role in articulatory recoding and top–down phonological control (Démonet et al., 1992, Poldrack et al., 1999, Tan et al., 2005). Functional connectivity between left IFG and left STG was also positively related to TOWRE, indicating that skilled readers exhibit stronger coordination between frontal and temporal language-related regions (Woolnough et al., 2023). Together, these fMRI findings suggest that both localized activation and interregional coupling within the frontotemporal network are associated with reading proficiency.

Electrophysiological markers provided convergent but differentiated insight into reading variability. The P200 component, observed over left frontotemporal electrodes, was associated with reading fluency and spelling, suggesting that early, rapid phonological binding supports both speed and precision of print–speech integration. Time–frequency analyses likewise revealed that greater theta-band (4–7 Hz) power for AV relative to unimodal stimulation was associated with reading efficiency and fluency. Because theta oscillations have been broadly linked to phonological working memory, temporal coordination, and cross-modal binding (Bastiaansen and Hagoort, 2006, Obleser and Kayser, 2019), this pattern suggests that stronger low-frequency synchronization may support fluent sequencing and retrieval of phonological representations. Taken together, these findings suggest that successful reading depends on the coordinated operation of frontotemporal mechanisms at multiple stages: automatic sensory convergence in temporal cortex supporting orthographic learning; rapid phonological binding facilitating decoding; and individualized recruitment of frontal control processes contributing to evaluation of print–speech consistency. Rather than reflecting redundant processes, these neural markers capture complementary dimensions of reading, illustrating how distinct stages of AV integration jointly contribute to skilled reading.

### 4.3 Multimodal (fMRI–EEG) contributions to reading

The present findings demonstrated that combining fMRI and EEG measures substantially improved the prediction of individual differences in reading-related performance. Across the three behavioral outcomes—reading efficiency, reading fluency, and spelling—multimodal models explained more variance than the fMRI-only models, underscoring the complementary value of spatially and temporally resolved neural indices.

For reading efficiency, both fMRI and EEG predictors contributed unique variance. Stronger IFG–STG functional connectivity and greater congruency effects in IFG and STG were associated with better reading efficiency, consistent with more effective phonological–orthographic mapping within the frontotemporal reading network. Enhanced theta activity also predicted higher reading efficiency, suggesting that low-frequency oscillations coordinate temporal dynamics supporting phonological integration. When all neural predictors were entered into the same model, some fMRI effects that were significant in unimodal analyses became nonsignificant, indicating shared variance between fMRI and EEG. This shared variance likely reflects overlapping sensitivity to core phonological decoding processes: both fMRI and EEG capture how efficiently the left frontotemporal network links print to sound—fMRI indexing its spatial integration, and EEG indexing its temporal synchronization.

For reading fluency, the improvement from adding EEG measures was moderate and driven primarily by theta power, while none of the fMRI predictors contributed unique variance. This pattern suggests that although the left frontotemporal is important for phonological decoding, fluency may depend more on neural timing—how rapidly and rhythmically phonological representations are accessed and sequenced during reading. The unique variance contributed by EEG likely reflects individual differences in temporal efficiency and phase alignment across reading-related regions—features that are not well captured by the slower hemodynamic signal. For spelling, the P200 amplitude was the only significant predictor, indexing early visual–phonological encoding processes for letter–sound integration. Although fMRI measures explained some variance independently, their contributions became nonsignificant in the multimodal model, again reflecting partial overlap between fMRI and EEG signals. This common variance may represent shared sensitivity to early phonological-orthographic mapping within similar cortical circuit, whereas the unique variance of the P200 likely reflects rapid perceptual encoding.

### 4.4 Limitations and future directions

There were several limitations of this study. First, the relatively long fMRI session (∼40 min) may have introduced fatigue effects in this young sample, although the simple task demands and frequent catch trials were designed to maintain engagement. Second,

EEG and fMRI data were collected in separate sessions, precluding direct joint modeling analyses (e.g., EEG-informed fMRI) that could link temporal dynamics to their spatial generators. Future research employing simultaneous EEG-fMRI acquisition would be better positioned to examine joint modeling approaches and establish direct correspondence between EEG temporal signatures and fMRI-identified cortical sources. Third, the EEG paradigm included only AV congruent trials, whereas fMRI included both congruent and incongruent trials, precluding direct cross-modal comparison of congruency effects. Future studies using fully matched EEG–fMRI paradigms that include both congruent and incongruent audiovisual conditions will be needed to enable more direct comparisons between these two neural modalities. Fourth, the absence of congruency effects for single letters may be partially attributable to our use of broad anatomical ROIs based on the AAL atlas. More focal phoneme-sensitive subregions within the posterior STG/STS, which have been shown to be particularly sensitive to letter-speech sound congruency (van Atteveldt et al., 2004), may have been diluted within these larger ROIs. Future studies could employ functionally defined ROIs based on independent localizers or meta-analytic coordinates to examine whether more focal regions within these larger anatomical structures show different effects. Finally, fMRI/EEG and behavioral data were collected in separate sessions with an average interval of 18 days. Future research should aim to minimize this interval or collect data within a single session where feasible.

## 5 Conclusion

This study combined fMRI and EEG to examine the spatiotemporal neural underpinnings of print–speech integration and their associations with reading-related skills. Together, the two modalities uncovered a coordinated left frontotemporal system, centered on STG and with skill-dependent engagement of IFG, that linked spatially distributed and temporally synchronized processes for print–speech integration. Different neural metrics from fMRI and EEG were differentially associated with reading efficiency, reading fluency, and spelling. Multimodal models accounted for greater variance in reading skills than unimodal models, highlighting that efficient reading relies on the precise temporal coordination of distributed neural networks.

## CRediT authorship contribution statement

**Jinglei Ren:** Writing – review & editing, Writing – original draft, Visualization, Validation, Supervision, Software, Project administration, Methodology, Investigation, Formal analysis, Conceptualization. **Haiwa Wang:** Writing – review & editing, Writing – original draft, Visualization, Validation, Software, Project administration, Methodology, Investigation, Formal analysis, Data curation. **Nicole Landi:** Writing – review & editing, Supervision, Resources, Methodology, Conceptualization. **Joanisse Marc:** Writing – review & editing, Visualization, Supervision. **Kenneth Pugh:** Writing – review & editing, Supervision, Software, Resources, Methodology, Investigation, Funding acquisition, Conceptualization. **Fumiko Hoeft:** Writing – review & editing, Supervision, Software, Resources, Methodology, Investigation, Funding acquisition, Conceptualization. **Roeland Hancock:** Writing – review & editing, Supervision, Resources, Methodology, Investigation, Conceptualization. **Vincent Gracco:** Writing – review & editing, Supervision. **Dan Kleinman:** Writing – review & editing, Supervision. **Kelly Mahaffy:** Writing – review & editing, Resources, Project administration, Methodology. **Aahana Bajracharya:** Writing – review & editing.

## Declaration of Competing Interest

The authors declare that they have no known competing financial interests or personal relationships that could have appeared to influence the work reported in this paper.

## Data Availability

Data will be made available on request. All data can be obtained from the corresponding authors upon request.

## Footnotes

Appendix A Supplementary data associated with this article can be found in the online version at doi:10.1016/j.dcn.2026.101729.
