# Supplementary material for: Audiovisual integration in reading among school-aged children: Evidence from combined fMRI and EEG
Source: Dev Cogn Neurosci. 2026 Apr 28;79:101729. doi: 10.1016/j.dcn.2026.101729 (PMC13147406; doi:10.1016/j.dcn.2026.101729)
Supplement: Supplementary file 1 — Supplementary material [file mmc1.docx]

**S1. Experimental Design and Stimuli Details**

**S1.1 Visual Stimuli**

Visual stimuli were presented in lowercase Arial font, size 36 pt, in black text on a white background. Letters were presented in lowercase throughout the experiment.

Nonword (CVC) string generation. Nonword stimuli were constructed as pronounceable consonant-vowel-consonant (CVC) strings following English phonotactic constraints. Consonants were selected from the set of high-frequency English consonants, and vowels included the five standard English vowels (a, e, i, o, u). Letter combinations were constrained to form phonotactically legal sequences in English (e.g., mog, bab, wap), avoiding illegal onset or coda clusters. All nonwords were verified to be pronounceable and not homophonous with real English words.

Visual stimulus presentation. During fMRI scanning, visual stimuli were presented via an MRI-compatible LCD projector and viewed through a mirror mounted on the head coil. The screen was positioned approximately 110 cm from participants' eyes, and the word stimuli subtended approximately 2.5°–3.0° of visual angle (depending on word length). During EEG recording, visual stimuli were presented on a 24-inch LCD monitor positioned approximately 70 cm from participants, with stimuli subtending approximately 2.8° of visual angle.

Cross-session consistency. Stimulus parameters (size, duration, font, color, timing) were matched across EEG and fMRI sessions. Both sessions used the same stimulus presentation software (E-Prime 2.0) to ensure precise timing and identical stimulus delivery.

**S1.2 Auditory Stimuli**

Auditory stimuli generation. Spoken letter names and nonwords were recorded by a female native American English speaker. All auditory stimuli were edited in Praat. Recordings were RMS-normalized to ensure consistent perceived loudness across stimuli and trimmed to remove silence at onset and offset.

Stimulus volume was initially set to a standardized preset level. For EEG sessions, Participants were asked at the start of each session whether the volume was too loud or too soft, and individual adjustments were made to ensure comfortable listening. For fMRI sessions, after the initial setup, participants were asked to confirm they could clearly hear the stimuli over the scanner noise; volume was increased if necessary to ensure audibility.

**S1.3 Task Design and Item Repetition**

Item repetition structure. Individual stimulus items were repeated across trials within each session to obtain sufficient signal for neural analyses. In the fMRI session, each unique letter (single-letter task) or nonword (multi-letter task) was presented 3 times across conditions. In the EEG session, item repetition was slightly higher due to the increased number of trials per condition, with each unique item presented 4 times. The repetition structure was balanced across conditions within each paradigm, ensuring that any repetition-related effects would affect all conditions equally and would not confound the contrasts of interest. Although the absolute number of repetitions differed slightly between fMRI and EEG sessions due to differences in trial counts and condition structure, the relative repetition balance across conditions was maintained in both paradigms.

Trial randomization. Trials were presented in a fully randomized order within each task for both fMRI and EEG sessions. Visual, auditory, and audiovisual trials were intermixed randomly rather than presented in blocks, reducing anticipatory effects and ensuring that condition-related neural responses were not confounded by temporal position or expectation. A unique randomization sequence was generated for each participant.

Stimulus matching across conditions. Grapheme and phoneme occurrences were matched across auditory, visual, and audiovisual conditions. For both single-letter and CVC tasks, the same set of stimulus items appeared in each modality condition (auditory, visual, AV congruent), ensuring equivalent frequency distributions of individual graphemes and phonemes across conditions. In the AV incongruent condition (fMRI only), visual and auditory stimuli were mismatched but drawn from the same stimulus pool, with no shared graphemes or phonemes between modalities (e.g., hearing "rud" while seeing "wap") to maximize mismatch detection.

Stimulus selection was guided by the following rationale: (1) phonotactic legality, all CVC nonwords conformed to English phonotactic constraints to ensure pronounceability; (2) non-lexicality, all CVCs were verified as nonwords and not homophonous with real English words, isolating sublexical orthographic-phonological mapping from semantic processing; (3) letter frequency, high-frequency consonants and vowels were selected to ensure familiarity for 7–10-year-old participants; and (4) balanced representation, each grapheme and phoneme appeared with approximately equal frequency across the stimulus set to prevent item-specific effects from driving condition differences.

**S2. Supplementary analyses**

**S2.1 fMRI and EEG behavioral performance**

Participants demonstrated high accuracy on catch trials across both tasks (single-letter: *M* = 91%, *SD* = 11%; multi-letter: *M* = 86%, *SD* = 13%). Mean reaction times were 652 ms (*SD* = 159 ms) for single-letter and 672 ms (*SD* = 178 ms) for multi-letter detection. Similarly, accuracy was high in the EEG session (single-letter: *M* = 88%, *SD* = 14%; multi-letter: *M* = 87%, *SD* = 12%), with mean reaction times of 678 ms (*SD* =189 ms) and 696 ms (*SD* = 192 ms), respectively.

Paired t-tests comparing performance between EEG and fMRI sessions revealed no significant differences in accuracy or reaction time (*ps* >.30). A repeated-measures ANOVA examining effects of condition (visual, auditory, AV congruent, [AV incongruent for fMRI]) and letter type (single vs. multi) on accuracy revealed no significant effects (*ps* >.21). These results confirm that participants were attentive and engaged across both sessions and all conditions, supporting the validity of the neural findings.

**S2.2 fMRI site effects**

We also conducted additional analyses to address potential site effects in the fMRI data. 1. Site as a nuisance covariate: We re-ran whole-brain models analyses including site (Yale vs. UConn) as a nuisance covariate in the second-level GLM models. The pattern of results remained consistent with our original findings. Specifically: The additive enhancement effect (AV > (A+V)/2) in bilateral STG and Heschl's gyrus remained significant (left STG: *β* = 0.48, *p*-FWE = 0.026; right STG: *β* = 0.45, *p*-FWE = 0.031; left Heschl's gyrus: *β* = 0.42, *p*-FWE = 0.028; right Heschl's gyrus: *β* = 0.39, *p*-FWE = 0.031). The congruency effect (incongruent > congruent) for multi-letter stimuli in left IFG and left STG remained significant (left IFG, triangularis: *β* = -0.39, *p*-FWE = 0.038; left IFG, orbitalis: *β* = -0.43, *p*-FWE = 0.020; left STG: *β* = -0.42, *p*-FWE = 0.028; right MTG: *β* = -0.38, *p*-FWE = 0.037). 2. Site × effect-of-interest interactions: We tested whether site moderated any of our effects of interest by including site × condition interaction terms in our models. No significant site × modality interactions were observed for the additive enhancement contrast (all *ps* > .35). Similarly, no significant site × congruency interactions emerged (all *ps* > .40). These null interactions indicate that the neural effects were comparable across the two scanning sites.

**S2.3 Missing data analyses**

Given the substantial imbalance in sample sizes between the two groups (16 vs. 56), we report descriptive statistics rather than inferential tests. Descriptive statistics indicated comparable characteristics between the two groups. For age, the excluded group (*M* = 8.76, *SD* = 1.14) was similar to the retained group (*M* = 8.90, *SD* = 1.02). The sex distribution was also comparable, with 6 females and 10 males in the excluded group versus 24 females and 32 males in the retained group. For nonverbal intelligence (WASI Performance IQ), the excluded group (*M* = 92.40, *SD* = 14.23) showed similar scores to the retained group (*M* = 94.68, *SD* = 15.51). Therefore, the data were missing at random, and no systematic bias was involved.

**S2.4 Age effect analyses**

We conducted exploratory moderation analyses by adding interaction terms (age × neural index) to our brain–behavior regression models. Specifically, we tested whether age moderated the associations between each significant neural predictor (fMRI: additive enhancement in left STG, congruency effects in left IFG and left STG, IFG–STG connectivity; EEG: P200 amplitude, theta power) and reading outcomes (TOWRE, reading fluency, spelling). Results indicated that none of the age × neural index interaction terms reached statistical significance (all *ps* > .25), suggesting that the brain–behavior relationships we observed were consistent across the 7–10 year age range in our sample. This is consistent with prior work suggesting that the core neural architecture supporting print–speech integration is established by early elementary school, even as reading skills continue to develop (Brem et al., 2010; Maurer et al., 2006).


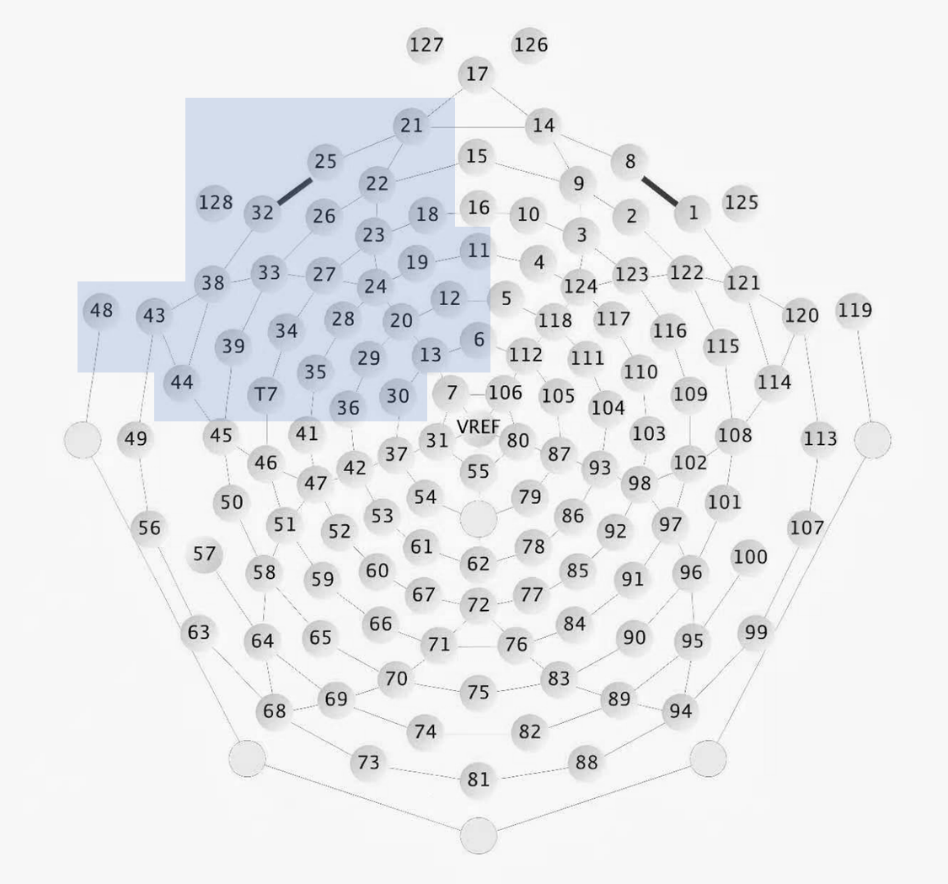


**eFigure 1.** Layout of the HydroCel Geodesic Sensor Net (128 channels). Blue-highlighted electrodes (E6, E7, E11, E12, E13, E18, E19, E20, E21, E22, E23, E24, E25, E26, E27, E28, E29, E30, E32, E33, E34, E35, E36, E38, E39, E43, E44, E48, E128) denote the channels used for ERP analyses, covering the left frontal and left temporal regions.


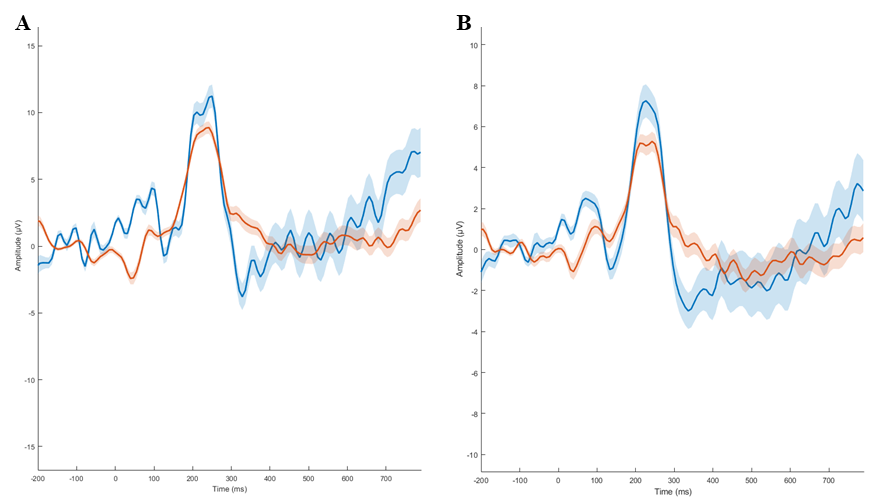


**eFigure 2.** Average waveforms for AV and (A+V)/2 conditions across different tasks. A. Average waveforms for single-letter tasks. B. Average waveforms for multi-letter tasks.


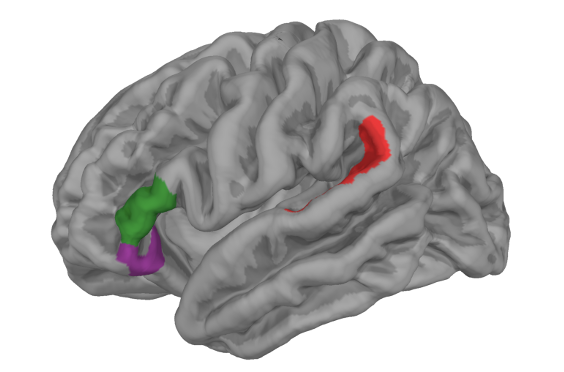
 **
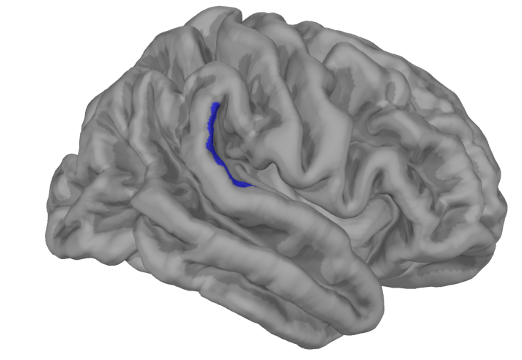

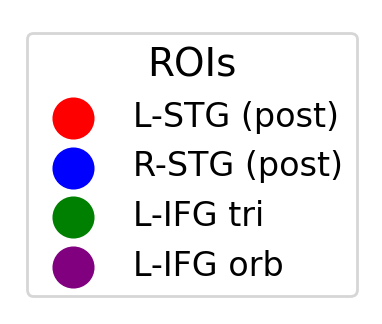
**

**eFigure 3.** Regions of Interest for Functional Connectivity Analysis. Anatomically defined ROIs displayed on the fsaverage template brain. Left hemisphere: left posterior superior temporal gyrus (L-pSTG, red), left inferior frontal gyrus pars triangularis (L-IFGtri, green), and left inferior frontal gyrus pars orbitalis (L-IFGorb, purple). Right hemisphere: right posterior superior temporal gyrus (R-pSTG, blue).

**References**

Brem, S., Bach, S., Kucian, K., Guttorm, T. K., Martin, E., Lyytinen, H., Brandeis, D., & Richardson, U. (2010). Brain sensitivity to print emerges when children learn letter-speech sound correspondences. *Proceedings of the National Academy of Sciences of the United States of America, 107*(17), 7939–7944. <https://doi.org/10.1073/pnas.0904402107>

Maurer, U., Brem, S., Kranz, F., Bucher, K., Benz, R., Halder, P., Steinhausen, H. C., & Brandeis, D. (2006). Coarse neural tuning for print peaks when children learn to read. *NeuroImage, 33*(2), 749–758. <https://doi.org/10.1016/j.neuroimage.2006.06.025>
